# A Role for the Chicken Interferon-Stimulated Gene *CMPK2* in the Host Response Against Virus Infection

**DOI:** 10.3389/fmicb.2022.874331

**Published:** 2022-05-11

**Authors:** Xin Li, Yiyi Feng, Weiwei Liu, Lei Tan, Yingjie Sun, Cuiping Song, Ying Liao, Chenggang Xu, Tao Ren, Chan Ding, Xusheng Qiu

**Affiliations:** ^1^Shanghai Veterinary Research Institute, Chinese Academy of Agricultural Sciences, Shanghai, China; ^2^College of Veterinary Medicine, South China Agricultural University, Guangzhou, China; ^3^Key Laboratory of Animal Infectious Diseases, Yangzhou University, Yangzhou, China; ^4^Shanghai Key Laboratory of Veterinary Biotechnology, Shanghai, China; ^5^Jiangsu Co-innovation Center for Prevention and Control of Important Animal Infectious Diseases and Zoonoses, Yangzhou University, Yangzhou, China

**Keywords:** chCMPK2, H9N2, IFN, anti-viral innate immunity, thymidylate kinase

## Abstract

Virus infection can lead to the production of interferon, which activates the JAK/STAT pathway and induces the expression of multiple downstream interferon-stimulated genes (ISGs) to achieve their antiviral function. Cytidine/uridine monophosphate kinase 2 (*CMPK2*) gene has been identified as an ISG in human and fish, and is also known as a rate-limiting enzyme in mitochondria to maintain intracellular UTP/CTP levels, which is necessary for *de novo* mitochondrial DNA synthesis. By mining previous microarray data, it was found that both Avian Influenza Virus (AIV) and Newcastle Disease Virus (NDV) infection can lead to the significant upregulation of chicken *CMPK2* gene. However, little is known about the function of *CMPK2* gene in chickens. In the present study, the open reading frame (ORF) of chicken CMPK2 (chCMPK2) was cloned from DF-1, a chicken embryo fibroblasts cell line, and subjected to further analysis. Sequence analysis showed that chCMPK2 shared high similarity in amino acid with CMPK2 sequences from all the other species, especially reptiles. A thymidylate kinase (TMK) domain was identified in the C-terminus of chCMPK2, which is highly conserved among all species. *In vitro*, AIV infection induced significant increases in chCMPK2 expression in DF-1, HD11, and the chicken embryonic fibroblasts (CEF), while obvious increase only detected in DF-1 cells and CEF cells after NDV infection. *In vivo*, the expression levels of *chCMPK2* were up-regulated in several tissues from AIV infected chickens, especially the brain, spleen, bursa, kidney, intestine, heart and thymus, and notable increase of *chCMPK2* was detected in the bursa, kidney, duodenum, lung, heart, and thymus during NDV infection. Here, using MDA5 and IFN-β knockdown cells, we demonstrated that as a novel ISG, chCMPK2 could be regulated by the MDA5/IFN-β pathway. The high expression level of exogenous chCMPK2 displayed inhibitory effects on AIV and NDV as well as reduced viral RNA in infected cells. We further demonstrated that Asp135, a key site on the TMK catalytic domain, was identified as critical for the antiviral activities of chCMPK2. Taken together, these data demonstrated that chCMPK2 is involved in the chicken immune system and may play important roles in host anti-viral responses.

## Introduction

Humans and animals are under continual attack from invading microbes, and innate immunity is the first-line host defense against those invading pathogens, including viruses. As a part of host innate immunity, pattern recognition receptors (PRRs) can recognize viral pathogen-associated molecular patterns, and activate several antiviral signaling cascades, such as type I and type III interferons (IFNs), chemokines, and pro-inflammatory cytokines (Carty et al., 2021). Secreted type I and type III IFNs induce the expression of multiple IFN-stimulated genes (ISGs) through the JAK/STAT pathway, which exert direct and powerful antiviral effects (Schoggins, 2019).

Avian Influenza Virus (AIV) and Newcastle Disease Virus (NDV) infections cause serious diseases in birds and pose major challenges to the global poultry industry (Li et al., 2019; Gong et al., 2021). Investigating the antiviral targets against those pathogens will be helpful for the prevention of transmission of avian infectious diseases and solving public health issues. It is now clear that IFNs activated by host RLRs (Retinoic acid-inducible gene I-like receptors) play a central role in the process of both anti-AIV and anti-NDV (Liniger et al., 2012; Xu et al., 2015; Han et al., 2019). The retinoic acid-inducible gene I (RIG-I) and Melanoma Differentiation-Associated Protein 5 (MDA5) are two key proteins of PRRs that are present in the cytoplasm, which act as sensors of viral RNA to mediate innate antiviral immune responses (Yoneyama and Fujita, 2007; Zou et al., 2009; Brisse and Ly, 2019). Although RIG-I and MDA5 are conserved among vertebrates, RIG-I is apparently absent in chickens but present in ducks (Zou et al., 2009; Barber et al., 2010). In absence of RIG-I, AIV, NDV and other avian viruses can still stimulate significant type I IFN responses in DF-1 and HD11 chicken cell lines through chicken MDA5 instead (Karpala et al., 2011; Liniger et al., 2012).

Cytidine/uridine monophosphate kinase 2 (CMPK2), also known as TYKi/TMPK2, has been identified as a MDA5-associated ISG in human and fish (Collins et al., 2007) and also proven to be associated with host inflammation responses (Zhong et al., 2018; Qi et al., 2021). In mammal, the expression of CMPK2 was ubiquitously distributed in different tissues, and significantly high levels were observed in the liver, pancreas, placenta, lung and heart and THP1 monocytic leukemia cells as well as human monocytes (Chen et al., 2008). Human CMPK2, as a IRF3-type IFNAR dependent cytokine, induced explicitly by poly(I:C) and several viruses (Kim et al., 2021). In term of virus infection, human CMPK2 was significantly increased after the infection of viruses, such as Hepatitis E virus (HEV) and Porcine reproductive and respiratory syndrome (PRRS) virus (Zhang et al., 2014; Kommadath et al., 2017). Further data of Chen et al. proved that the upregulation of CMPK2 is associated with monocyte/macrophage differentiation which was induced by PMA which pointed that CMPK2 may represent a mechanism for maintaining cell biogenesis. In 2019, Collins et al. isolated fish CMPK2 from salmon and found that fish CMPK2 can also be upregulated upon LPS (bacterial mimic) and poly(I:C; viral mimic) stimulation (Liu et al., 2019).

CMPK2 belongs to a nucleoside monophosphate kinase family which is involved in phosphorylation of dUMP, dCMP, CMP, and UMP using ATP as a phosphate donor (Xu et al., 2008; Gul et al., 2020). It was found that the best natural substrate for CMPK2-mediated phosphorylation was dUMP, and followed by dCMP, CMP, and UMP (Xu et al., 2008). Previous research has proven that a nucleoside monophosphate kinase domain, also known as thymidylate kinase (TMK) domain, was contained in the C-terminus of CMPK2 (Chen et al., 2008; Xu et al., 2008); while a mitochondrial targeting signal was located in its N-terminal domain. CMPK2 is located in the mitochondria of mammal cells, and also known as a rate-limiting enzyme in mitochondria to maintain intracellular UTP/CTP levels, which is necessary for *de novo* mitochondrial DNA synthesis (mtDNA; Feng et al., 2021). Zhong et al. (2018) showed that the catalytic activity of CMPK2 in mitochondria was essential for NLRP3-dependent caspase-1 activation and interleukin (IL)-1β production. In a study of the therapeutic effects of cannabidiol (CBD) on oral ulcer, it was found that CBD accelerated oral ulcer recovery by inhibiting CMPK2-mediated NLRP3 inflammasome activation and pyroptosis (Qi et al., 2021).

Previous results revealed that CMPK2 may play a role in the innate immune and inflammation responses against viruses in a variety of ways (El-Diwany et al., 2018; Feng et al., 2021). In a recent study, El-Diwany et al. (2018) showed that CMPK2 was upregulated in activated CD4^+^ T cells after HIV patients were treated with IFN-α2b; treatment with type I IFN reduced plasma HIV RNA levels, but this effect was inhibited when CMPK2 was knocked down. CMPK2 maintains adequate substrate levels for viperin-mediated production of ddhCTP, which can directly inhibit replication of ZIKA virus *in vivo* (Gizzi et al., 2018). Moreover, the antiviral activities of fish CMPK2 during Spring viraemia of carp virus (SVCV) infection were confirmed by overexpression and RNA interference assays (Liu et al., 2019).

Since little is currently known about the role of CMPK2 in chickens, the open reading frame (ORF) of chicken *CMPK2* (*chCMPK2*) gene was amplified in this study and subjected to further researches for understanding the related molecular mechanism of chicken immune response against avian viral diseases. We found that avian virus infection could induce the high expression of *chCMPK2* gene, and propagation of AIV H9N2 and NDV was significantly blocked by chCMPK2 in a dose-dependent manner. We further demonstrated that alanine substitution on D135, a critical residue for the TMK domain, could abolish the antiviral effects of chCMPK2. Our findings could facilitate the development of effective measures to enhance immunity against RNA viruses in poultry.

## Materials and Methods

### Cells and Viruses

Chicken fibroblast DF-1, macrophage-like HD11, and B-cell lymphoma DT40 cell lines were obtained from the American Type Culture Collection and cultured in Dulbecco’s Modified Eagle’s Medium (DMEM; Gibco, Grand Island, NY, United States) or RPMI 1640 medium (Gibco) containing 10% fetal bovine serum (FBS, Gibco) at 37°C and 5% CO_2_. Chicken embryo fibroblasts (CEFs) were prepared from 9- to 11-day-old specific pathogen-free (SPF) chicken embryonated eggs (MERIAL, Beijing, China), as previously described (Qiu et al., 2011).

Newcastle Disease Virus strains LaSota/46 and Mukteswar were obtained from the China Institute of Veterinary Drug Control (Beijing, China). AIV strain H9N2 (A/Chicken/Shanghai/010/2008) was isolated from ducks in Shanghai in 2008 (Cheng et al., 2015a). The 50% tissue culture infective dose (TCID_50_) for viruses were titrated onto DF-1 or MDCK cells using the Reed and Muench method. Virus titers were calculated by determining the dilution that yielded 50% of cells displaying cytopathic effects (Pizzi, 1950).

### Amplification of the Chicken *CMPK2* Gene

The *chCMPK2* amplification primers were designed according to the National Center for Biotechnology Information (NCBI) reference sequence (GenBank: XM_015284945.3). The *chCMPK2* cDNA was synthesized from total cellular RNA isolated from DF-1 cells. Then the entire ORF of the *chCMPK2* gene was amplified using the primers chCMPK2_F and chCMPK2_R (Supplementary Table S1, synthesized in Sangon Biotech Co.). The PCR conditions used were as follows: an initial denaturation step at 95°C for 3 min, followed by 30 cycles of 95°C for 15 s, 66°C for 30 s, and 72°C for 1 min and one final extension step at 72°C for 5 min. PCR products were purified and inserted into the pMD19T vector for Sanger sequencing.

### Plasmids and Transfection

The PCR products were inserted into the *EcoR*I/*BamHI* sites of the p3xFLAG-CMV™-14 (pCMV-Flag) vector to generate the pCMV-Flag-CMPK2 expression plasmid. Successful insertion was confirmed by sequencing. To verify the expression of the recombinant plasmid, HEK293T cells were transfected with pCMV-Flag-CMPK2 or p3xFLAG-CMV™-14 empty vector by Lipofectamine 2000 (Invitrogen, Carlsbad, CA, United States) in accordance with the manufacturer’s instructions when the cell monolayer reached 80–90% confluence. At 24 h post-transfection, the cells were collected for Western blot (WB) assay. The FLAG-labeled chCMPK2 protein was detected using an anti-FLAG antibody (Cell Signaling Technology, Danvers, MA, United States).

### Bioinformatics Analysis of chCMPK2

According to the sequencing results of *chCMPK2* and *CMPK2* sequences of other animals obtained from NCBI, the nucleotide sequences were aligned with CLUSTALW and then a phylogenetic tree was constructed based on the amino acid sequences according to the maximum likelihood algorithm by MEGA7.

The amino acids of these sequences were uploaded to the EMBL-EBI website^1^ for similarity analysis by using the multiple sequence alignment tools. The predicted human CMPK2 structure was obtained from the AlphaFold Protein Structure Database,^2^ based on which the 3D structure of chCMPK2 was constructed on the Swiss Model website.^3^ All structural annotations were generated using PyMOL (version 1.74, Schrödinger).

### Data Mining From GEO Database

Data of gene expression microarrays (GSE40100, GSE65231) were obtained from the GEO database. Differentially expressed genes (DEGs) were obtained by GEO2R using the cut-off standards |log_2_(fold change)| > 1, *p* < 0.05.

Heat maps plot was drawn using the “pheatmap” packages in R version 4.0.3. Venn analysis was performed using the jvenn website.^4^

### Preparation of Mouse Sera Against the chCMPK2 Protein

To test the chCMPK2 protein, mouse polyclonal serum against chCMPK2 was produced by the following steps. A highly conserved amino acid region (139–253) of chCMPK2 was chosen by aligning with the human and mouse CMPK2 amino acid sequences. This region was then cloned into pET28a (+) using *EcoR*I*/Sal*I sites to generate pET28a-chCMPK2. The recombinant chCMPK2 protein, which was labeled with a His tag, was expressed in *Escherichia coli* BL21 cells and column-purified as described in the pET system manual. Polyclonal antiserum against chCMPK2 was raised in 6-week-old BALB/c mice, as described previously (Qiu et al., 2016). To minimize mouse suffering and distress, all invasive manipulations were carried out under anesthesia using 1% sodium pentobarbital at a dose of 50 mg/kg body weight. No unexpected deaths occurred during this study. The mice were euthanized by CO_2_ inhalation for 5 min at the end of the study.

### Viral Infection, Total RNA Extraction, and Quantitative Real-Time PCR Assay

To measure the dynamic expression pattern of *chCMPK2* after virus infection in different cells, cells seeded in 12-well plates were infected with AIV H9N2 or NDV strain Mukteswar and LaSota strain. At 6 and 12 h post-infection (hpi), cells were harvested, stored in TRIzol reagent (Invitrogen, Carlsbad, CA, United States), and then subjected to RNA extraction according to the manufacturer’s instructions. Total cDNA was synthetized after RNA pellets were re-suspended in 50 μl RNase-free water and reverse transcribed with Oligo (dT) 18 Primer (Takara, Dalian, China) using Mo-MLV reverse transcriptase (Promega, Madison, WI, United States).

The cDNA of chicken tissues was collected in a previous study (Shi et al., 2020). SPF chickens were hatched from SPF eggs (MERIAL, Beijing, China) and randomly divided into uninfected and NDV-infected groups. At the age of 4 weeks, each chicken in the NDV-infected or H9N2-infected group was intramuscularly challenged with 0.2 ml containing 1 × 10^5^ EID_50_ NDV strain LaSota/46 or AIV H9N2. The uninfected control group was mock-infected with PBS. At 3 days post-infection (dpi), the organs from five chickens were collected, including the spleen, bursa, thymus, kidney, liver, lung, duodenum, intestine, brain, and heart.

The qRT-PCR analysis was performed to determine the expression levels of *chCMPK2* mRNA and viral RNA. The primers used for qRT-PCR are shown in Supplementary Table S2. *CMPK2* levels and the levels of RNA encoding H9N2 NP were measured at different hpi, and *CMPK2* mRNA levels were normalized to *β-actin*. The fold changes of relative mRNA levels were determined using the comparative 2^−ΔΔ*C*t^ method as previously described (Schmittgen and Livak, 2008).

### Western Blot Assay

To determine the expression levels of chCMPK2 and viral NP proteins (TransGen Biotech, China), cells were washed thoroughly in PBS twice and then lysed in 2× SDS loading buffer. Lysates were resolved by 10% SDS-PAGE, after which the proteins were transferred to nitrocellulose membranes (Whatman International, Ltd.), which were blocked for 2 h with 2% BSA in Tris-buffered saline with Tween 20 (TBST) at room temperature. Then the membranes were incubated with rabbit anti-β-actin, mouse anti-chCMPK2, and anti-NP, followed by incubation with HRP-conjugated secondary antibodies (Cell Signaling Technology) for 2 h at room temperature. The protein bands were visualized by enhanced chemiluminescence (Amersham Pharmacia Biotech, United Kingdom) using a Kodak imager (Carestream Health, Inc., Rochester, NY, United States).

### The chCMPK2 Knockdown by siRNA

*chCMPK2*-specific short interfering RNA (siRNA) and negative control (siNC) were designed and synthesized by Gene Pharma, Shanghai. The sequences of siCMPK2-1, siCMPK2-2, and siCMPK2-3 are shown in Supplementary Table S3. DF-1 cells were seeded into a 12-well plate and incubated for 12 h before transfection. Each well was washed twice with PBS and transfected with either 40 pmol siRNA mix or 40 pmol siNC with Lipofectamine 2000. At 36 h post-transfection, the cells were infected with AIV H9N2 at a multiplicity of infection (MOI) of 0.1. At 12 hpi, the cells were harvested to quantify the knockdown efficiency of *chCMPK2* by qRT-PCR and WB assays.

### Confocal Microscopy

DF-1 cells were seeded into 6-well glass bottom plates (Corning Incorporated, Corning, NY) the day before transfection. For mitochondrial colocalization analysis, cells were incubated with Mito-Tracker Red CMXRos (Beyotime Institute of Biotechnology, Jiangsu, China) for 30 min after transfection with pCMV-Flag-CMPK2 and pCMV-Flag for 36 h. After fixation with 4% formaldehyde, cells were permeabilized with 0.2% Triton X-100 and blocked in PBS with 3% BSA. Anti-Flag antibodies were used at a dilution of 1:500. Fluorophore-conjugated secondary antibodies and DAPI (4′,6-diamidino-2-phenylindole) were diluted 1:1,000 in PBS. Confocal laser scanning microscopy was performed with a Zeiss LSM510 confocal laser scanning microscope (Carl Zeiss Microimaging GmbH, Germany) fitted with a Plan Apochromat 63×/1.4 oil objective. Images were analyzed with Fiji software^5^ and ImageJ software. The experiments were repeated at least three times.

### Statistical Analysis

All experiments were performed in triplicate, and statistical analysis was conducted with GraphPad Prism 8 (GraphPad Software, San Diego, CA, United States). Data were analyzed with one-way analysis of variance (ANOVA) with Kruskal-Wallis comparison or two-way ANOVA with Tukey’s comparison. Statistical significance was defined as *p* < 0.05.

## Results

### Data Mining From GEO Database

**Figure 1:**
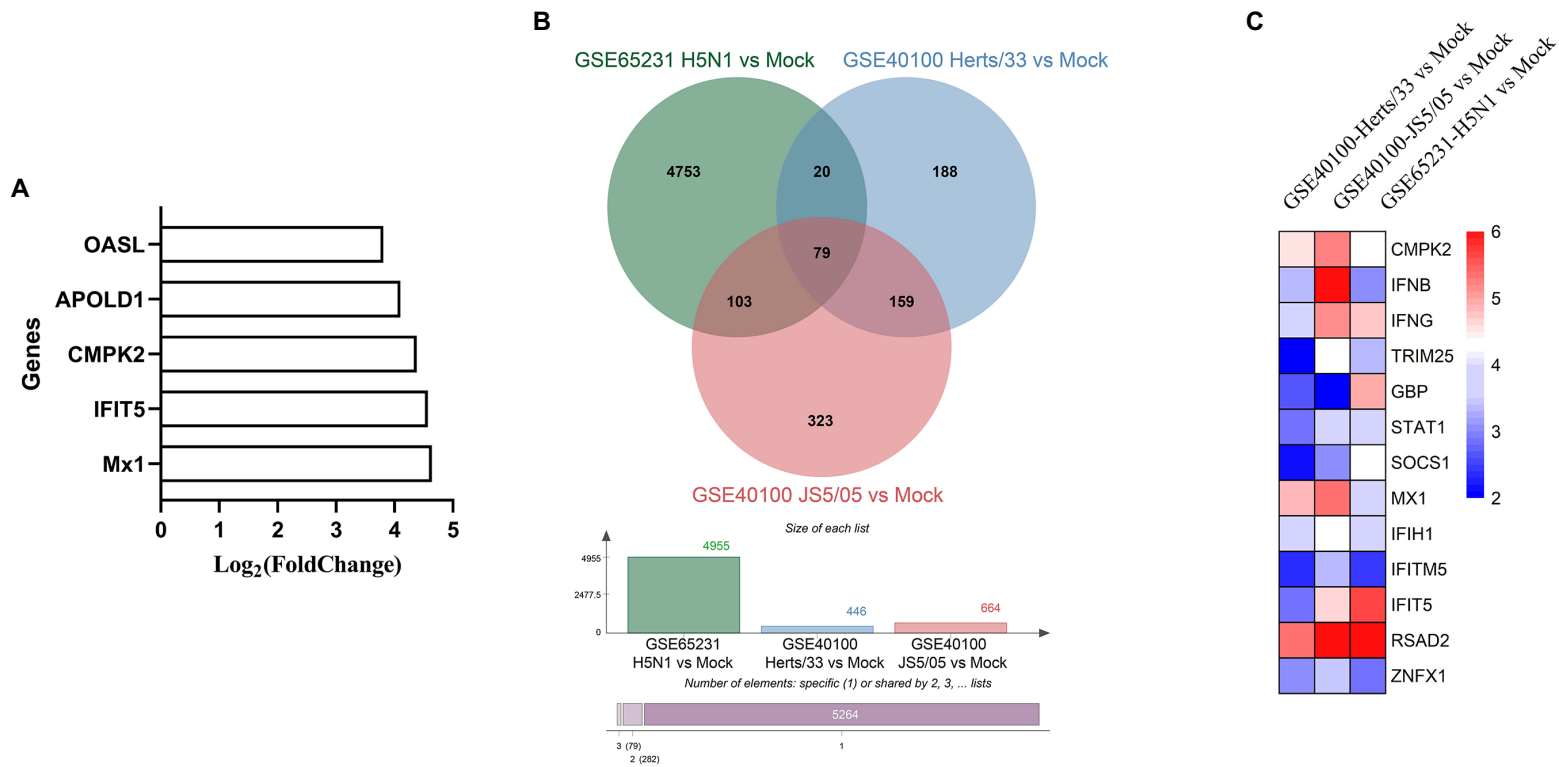
Differential expression analysis of CMPK2 in response to virus infection based on GEO database. **(A)** Histogram showing the top 5 upregulated genes according to a previous study in NDV-infected DF-1 cells. **(B)** Venn plot showing common upregulated genes with significant differential induction (Herts/33-, JS5-, or H5N1-infected versus uninfected). **(C)** Heat map showing fold change values after AIV H5N1 and NDV Herts/33 and JS5/05 infection of genes related to IFN and ISGs among the 79 common upregulated genes in **(B)**.

To identify undiscovered molecules involved in host innate immunity, we analyzed our previous transcriptomics data of NDV-infected DF-1 cells (Liu et al., 2018), and found that chicken *CMPK2* was notably upregulated as one of the top five upregulated host genes, including *OASL*, *APOLD1*, *IFIT5*, *Mx1*, indicating that the IFN response was apparently activated, in response to NDV strain Herts/33 (Figure 1A). We then analyzed two public data in GEO database: GSE65231 (gene expression profiling data of the lung of H5N1-infected chicken; Ranaware et al., 2016) and GSE40100 (gene expression profiling data of the spleen under NDV infection; Hu et al., 2015) and identified 79 overlapping upregulated genes (4,955 in GSE65231 H5N1 vs. Mock, 446 in GSE40100 Herts/33 vs. Mock, and 664 in GSE40100 JS5/05 vs. Mock) which is showed in Venn plot (Figure 1B). In these common upregulated genes, *CMPK2* ranks in the forefront with log_2_ (fold-change) values of 5.45, 4.336017, and 5.2694117. The genes related to IFN and ISGs, including *CMPK2* and several classic antiviral factors *Mx1*, *TRIM25*, and *RASD2*, among the top 20 are shown in a heat map (Figure 1C). Based on these data, we speculated that chicken CMPK2 may be involved in the immune response upon NDV and AIV infection.

### Molecular Cloning and Bioinformatics Analysis of chCMPK2

The cDNA from AIV H9N2-infected DF-1 cells was used as a template to amplify the *chCMPK2* gene. Our results showed that the chCMPK2 ORF consisted of 762 base pairs encoding 254 amino acids (Supplementary Figures S1A,B), which was the same as the reference sequence obtained from the chicken tissues (GenBank, accession No. XM_015284945.3). The molecular weight of chCMPK2 was around 33 kDa which was different from the predicted size of 28.03 kDa (Supplementary Figure S1C).

**Figure 2:**
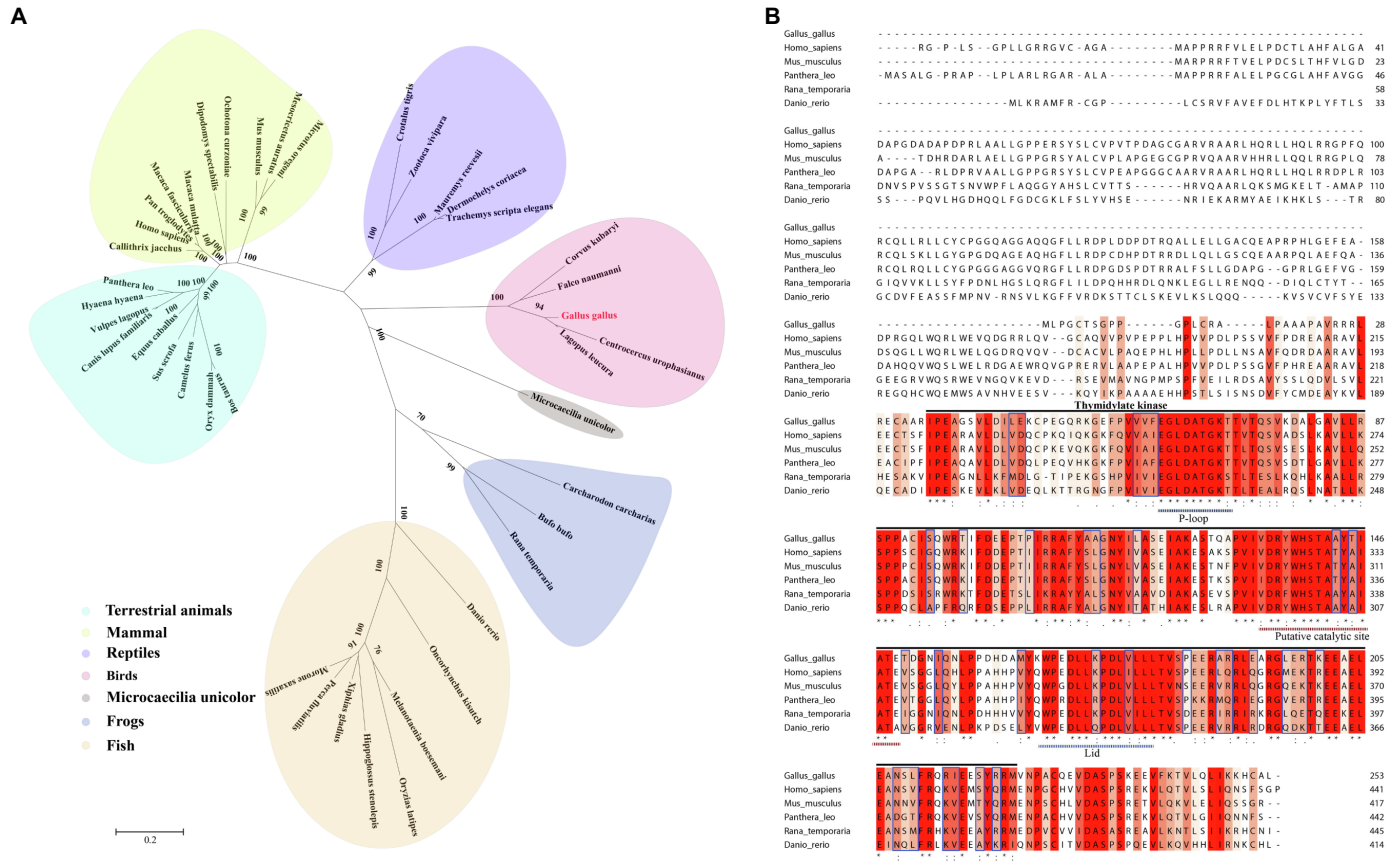
Bioinformatics analysis of chicken CMPK2. **(A)** Phylogenetic tree of CMPK2 using an amino acid sequence alignment among different species. Animals belonging to the same species are marked with the same color. **(B)** Alignment of *Gallus gallus* (XP_040523494.1), *Homo sapiens* (AAI41803.1), *Mus musculus* (AAH57565.1), *Panthera leo* (XP_042789682.1), *Rana temporaria* (XP_040204584.1), and *Danio rerio* (XP_699055.5) CMPK2 amino acid sequences using the Multiple Sequence Alignment tools on the EMBL-EBI website. Shading indicates sequence conservation, with deeper red indicating a higher degree of conservation. Conserved thymidylate kinase domain sequences were analyzed. At the bottom of the aligned sequences, “*” indicates fully conserved residues; “:” indicates identical or similar residues; “.” indicates relatively conserved residues. The P-loop, putative catalytic site, and lid motif are indicated by “…...”

Alignment and phylogenetic analysis of chCMPK2 with other CMPK2 amino acid from different species indicated that chCMPK2 was highly conserved among birds, such as *Centrocercus urophasianus*, *Lagopus leucura*, *Falco naumanni*, and *Corvus kubaryi*. CMPK2 from *Homo sapiens* and *Mus musculus* form a subclade and were distant from chicken CMPK2, which is obviously distant from reptile sequences (Figure 2A). According to the full-length amino acid sequence alignment between birds and other species, the amino acid homology between chicken CMPK2 and other species was 34.43–69.57% (Table 1). Further multiple alignment showed that chicken CMPK2 shared 70.74, 68.62, and 72.08% amino acid identity to *H. sapiens*, *M. musculus*, and *Panthera leo* in the TMK domain, also known as thymidine monophosphate kinase (TMPK), whose functional domain consists of a P-loop, a catalytic site, and a lid motif (Table 2; Chen et al., 2008; Xu et al., 2008). According to the sequence of human CMPK2, the P-loop, the putative catalytic site, and the lid domain are located at amino acids 63–72, 137–149, and 167–180 in chicken CMPK2 (Figure 2B). Multiple sequence alignment revealed that the three active domains of chCMPK2 TMK shared 81.25–100% amino acid identity with those of other species (Table 3), which may indicate that this domain is highly conserved in different species and plays an important role on the function of chCMPK2.

**Table 1:** Full-length CMPK2 amino acid sequence similarity among different species.

	Birds	Mammal	Terrestrial	Frogs	Reptiles	Fish	Microcaecilia unicolor
Birds (4)^*^	**>70.59**	60.08–63.85	60.16–63.85	54.37–56.63	57.71–69.57	34.43–53.36	61.04
Mammal (8)	–	**>70.22**	65.32–79.1	42.02–45.91	49.88–61.65	27.46–44.5	49.88–52.0
Terrestrial (6)	–	–	**>65.32**	41.69–47.24	49.88–61.79	29.23–43.8	50.37–53.44
Frogs (2)	–	–	–	**>54.21**	43.29–54.21	29.79–44.58	46.85–47.88
Reptiles (5)	–	–	–	–	**>57.57**	28.52–47.54	52.0–57.96
Fish (8)	–	–	–	–	–	**>39.79**	30.69–45.99
Microcaecilia unicolor	–	–	–	–	–	–	**100**

* *The number of reference sequences for each species was indicated in brackets. Homology within species are shown in bold*.

**Table 2:** TMK domain amino acid sequence similarity of CMPK2 among different species.

	*Gallus gallus*	*Homo sapiens*	*Mus musculus*	*Rana temporaria*	*Danio rerio*	*Panthera leo*
*Gallus gallus*	100	70.74	68.62	63.10	60.11	72.08
*Homo sapiens*	–	100	87.23	60.96	61.70	84.42
*Mus musculus*	–	–	100	61.50	62.23	83.12
*Rana temporaria*	–	–	–	100	59.89	58.82
*Danio rerio*	–	–	–	–	100	59.74
*Panthera leo*	–	–	–	–	–	100

**Table 3:** P-loop, catalytic site, and lid motif amino acid sequence similarity of CMPK2 among different species.

Species	Thymidylate kinase		
	P-loop	Putative catalytic site	Lid
*Gallus gallus*	100	100	100
*Homo sapiens*	100	87.50	92.86
*Mus musculus*	100	87.50	92.86
*Rana temporaria*	90	87.50	92.86
*Danio rerio*	100	87.50	92.86
*Panthera leo*	100	81.25	85.71

### Distribution of chCMPK2 in Cells and Tissues

**Figure 3:**
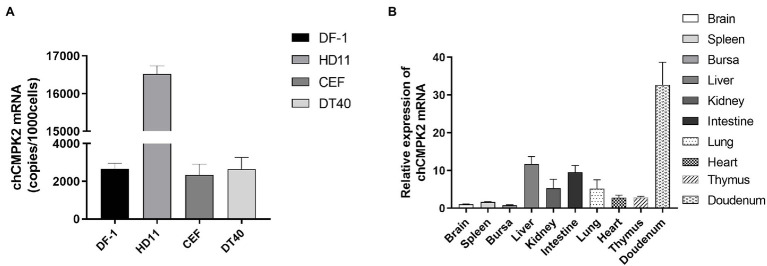
Distribution of chCMPK2 in different cells and chicken tissues. **(A)** DF-1 cells, HD11 cells, CEFs, and DT40 cells were seeded in 12-well plates until 90% confluence. The absolute quantification PCR was performed to detect *CMPK2* mRNA in all cell lines. *CMPK2* mRNA levels were showed by calculating copies per 1,000 cells (copies/1,000 cells). **(B)** *CMPK2* mRNA levels were measured in tissues (brain, spleen, bursa, liver, kidney, intestine, lung, heart, thymus, and duodenum) of SPF chicken.

A standard curve was generated using a tenfold serial dilution of the pCMV-Flag-CMPK2 plasmid, and the linear regression equation was *Y* = −5.251*X* + 51.495, with an *R*^2^ value of 0.998, indicating a strong linear correlation. As shown in Figure 3A, *chCMPK2* mRNA was highly expressed in the chicken macrophage line HD11; in contrast, lower expression of *chCMPK2* mRNA was observed in other chicken cells, including DF-1 cells, CEFs, and B lymphocyte line DT40. It suggested that *chCMPK2* mRNA was expressed in macrophages, which is consistent with the high expression of CMPK2 in human-derived macrophages cell line (THP-1; El-Diwany et al., 2018).

To obtain the expression profile of *chCMPK2* in 4-week-old SPF chickens, the abundance of *CMPK2* in organs was determined by normalizing the data with brain *via* using a comparative quantification method. The *chCMPK2* mRNA was detected in most tissues examined and showed tissue-specific differences. Expression levels were highest in the duodenum (*p* < 0.001), followed by the liver and the immune organs lung, intestine, and kidney, whereas relatively low expression was detected in the brain, spleen, and bursa (Figure 3B). Our results revealed that *chCMPK2* was highly expressed in the digestive system of chicken.

### Viral Infection Induces chCMPK2 Expression

To determine whether chCMPK2 could be expressed in protein level, mouse polyclonal antiserum against chCMPK2 was generated by immunizing mice with recombinant pET28a-chCMPK2 protein expressed and purified from *E. coli*. To test the validity and specificity of this serum, DF-1 cells transfected with pCMV-Flag-CMPK2 were used for WB assays at 24 h post-transfection. Anti-chCMPK2 serum specifically recognized Flag-tagged chicken CMPK2, which displayed similar-sized target bands to the anti-Flag antibodies (Supplementary Figures S1C,D).

**Figure 4:**
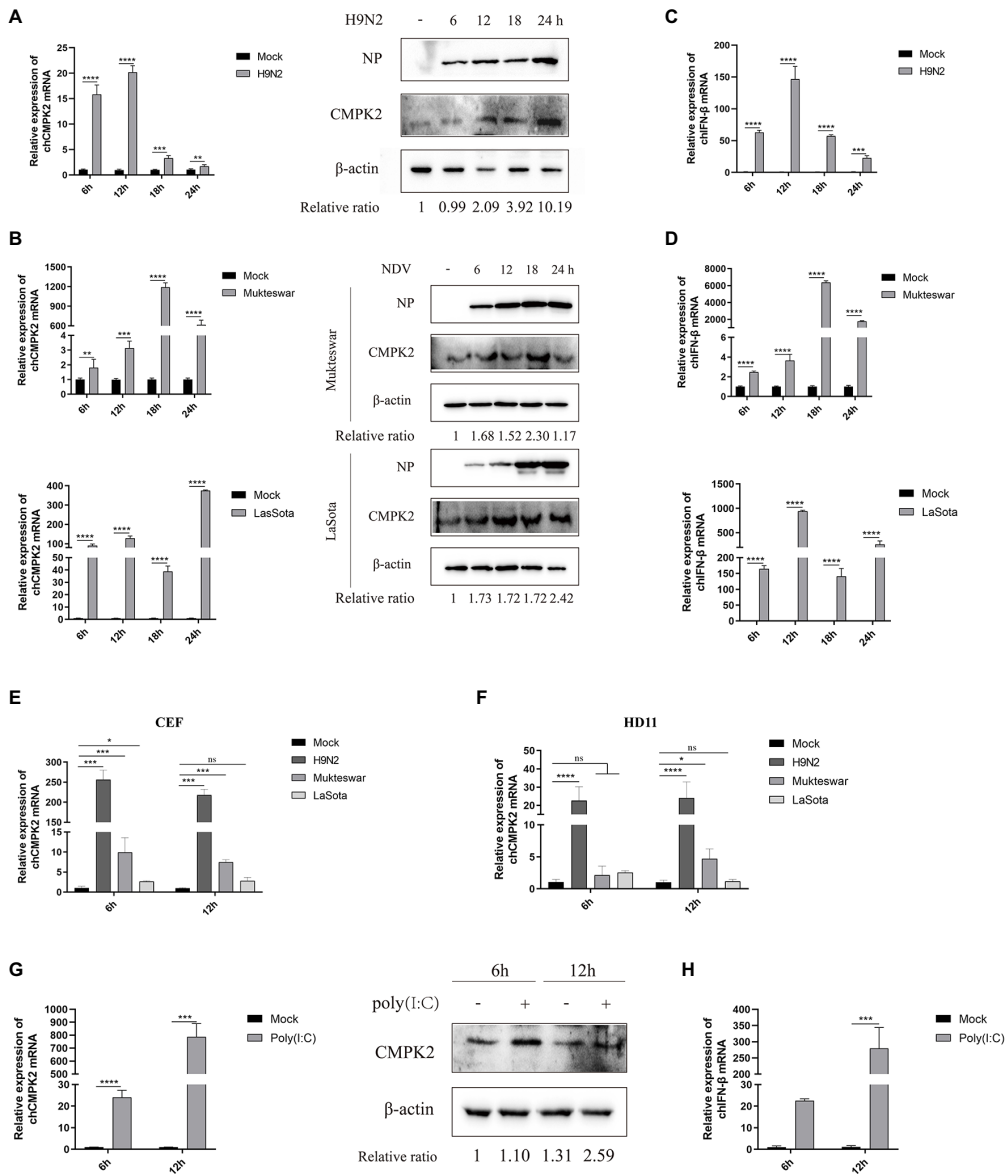
Viral infection induces chCMPK2 expression *in vitro*. **(A)** DF-1 cells were infected with H9N2 for 6, 12, 18, and 24 h and the mRNA and protein expression of CMPK2 were detected, as well as the expression of IFN-β was detected by qRT-PCR **(C)**. **(B)** DF-1 cells were infected with NDV Mukteswar and LaSota strains for 6, 12, 18, and 24 h and the mRNA and protein expression of CMPK2 were detected, as well as the expression of IFN-β was detected by qRT-PCR **(D)**. **(E,F)** HD11 cells, and CEFs were infected with AIV H9N2 and NDV Mukteswar, LaSota strains at an MOI of 1 for 6 and 12 h, and then the expression of *CMPK2* was detected by qRT-PCR. **(G)** DF-1 cells were treated with poly(I:C) at a concentration of 1 μg/ml for 6 and 12 h, and then the expression of CMPK2 was detected by qRT-PCR and WB while the expression of IFN-β was tested by qRT-PCR **(H)**. The gray intensity for each band of WB assays was measured, and the relative ratio of CMPK2 was determined from the equation (Sample^CMPK2^/Sample^actin^)/(Mock^CMPK2^/Mock^actin^) and marked at the bottom of each lane. Values represent the mean of the individual measurements in each sample ± SEM. ^*^p < 0.05, ^**^p < 0.01, ^***^p < 0.001, and ^****^p < 0.0001.

Previous studies have shown that CMPK2 was upregulated after stimulation with LPS, poly(I:C), Porcine Reproductive and Respiratory Syndrome Virus (PRRSV), and SVCV (Liu et al., 2019). The expression of chCMPK2 in response to chicken virus infection were determined in this study. The qRT-PCR assay results confirmed that the AIV H9N2 infection induced a substantial fold upregulation of the mRNA of *chCMPK2* at 6 and 12 hpi followed by the decrease at 18 and 24 hpi; while the expression of chCMPK2 protein increased from 6 to 24 hpi (Figure 4A). After NDV infection, the mRNA of *chCMPK2* increased from 6 to 18 hpi in the group of Mukteswar infection and from 6 to 24 hpi in the group of LaSota infection and the chCMPK2 protein levels showed similar trends (Figure 4B). Further, we evaluated *chCMPK2* expression in AIV and NDV-infected CEF and HD11 cells. The results showed that *chCMPK2* mRNA levels were significantly upregulated in CEF and HD11 cells during AIV H9N2 infection, while no significant increase was detected in NDV-infected HD11 cells (Figures 4E,F). The significant upregulated levels of mRNA and protein were obtained after stimulation with the double stranded RNA (dsRNA) analog poly(I:C; Figure 4G).

**Figure 5:**
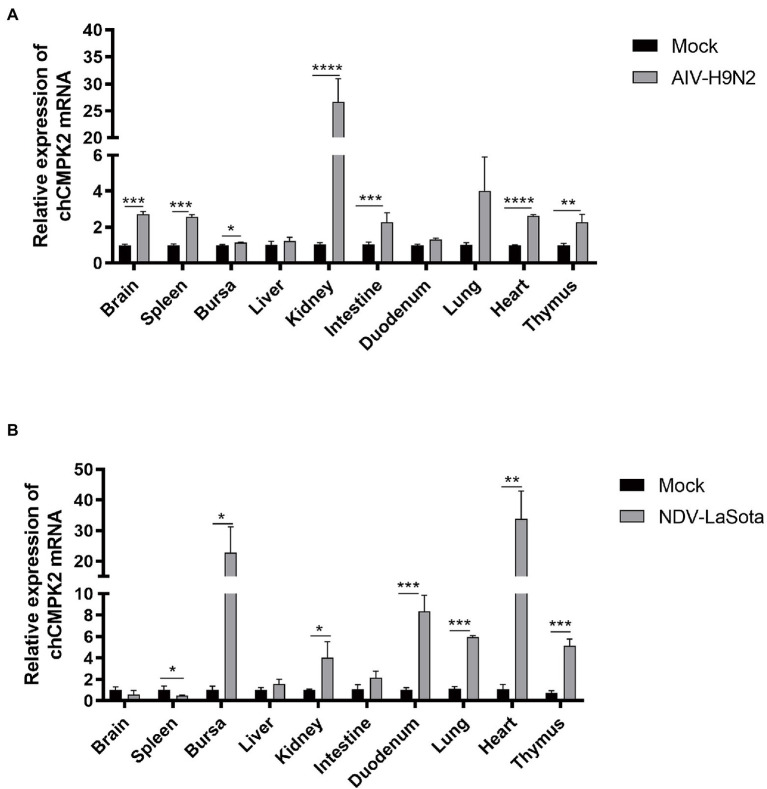
Viral infection induces chCMPK2 expression *in vivo*. SPF chickens were infected with AIV H9N2 **(A)** or NDV LaSota **(B)** at a dose of 0.2 ml containing 1 × 10^6^ EID_50_ or 1 × 10^5^ EID_50_, and the expression of *CMPK2* in different tissues (brain, spleen, bursa, liver, kidney, intestine, duodenum, lung, heart, and thymus) was detected by qRT-PCR at 3 dpi. Values represent the mean of the individual measurements in each sample ± SEM. ^*^p < 0.05, ^**^p < 0.01, ^***^p < 0.001, and ^****^p < 0.0001.

To detect the expression of ch*CMPK2 in vivo* during virus infection, the cDNAs samples of tissues, including brain, spleen, bursa, kidney, duodenum, lung, heart, and thymus were collected from chickens infected with AIV H9N2 or NDV LaSota at 3dpi in our previous studies, which was kept in −80°C (Shi et al., 2020). SPF chickens were infected by AIV H9N2 and NDV LaSota in a dose of 1 × 10^6^ EID_50_ and 1 × 10^5^ EID_50_, respectively, by intranasal inoculation. The high expression level of *CMPK2* in chicken intestine and the upregulation in immune-related tissues including thymus, spleen and lung after intranasal inoculation of AIV H9N2 were demonstrated by qRT-PCR (Figure 5A). In addition to the increased expression of *CMPK2* in immune organs, such as kidney, intestine and thymus, *CMPK2* levels still remained at a high level in the heart, bursa and duodenum after NDV LaSota infection (Figure 5B). Collectively, these findings suggest that chCMPK2 is involved in the host immune response against virus infection.

### The Upregulation of chCMPK2 Is Dependent on MDA5/IFN-β Pathway

**Figure 6:**
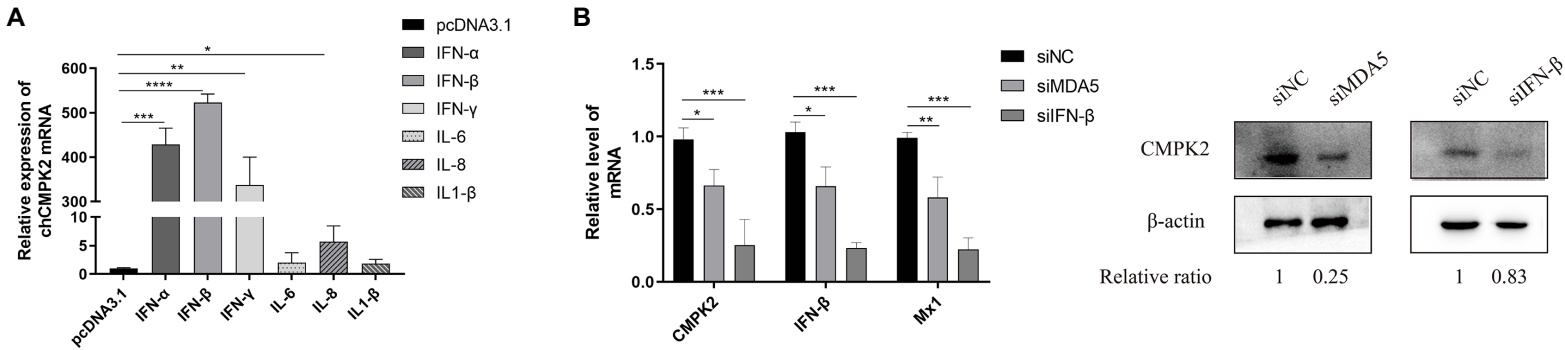
The expression of chCMPK2 is dependent on MDA5/IFN-β pathway. **(A)** The mRNA level of *CMPK2* in DF-1 cells was tested by qRT-PCR after transfection with pcDNA3.1 (control), IFN-α, IFN-β, IFN-γ, IL-6, IL-8, and IL-1β for 12 h. **(B)** DF-1 cells were treated with control siRNA (siNC) or small interfering RNA (siRNA) targeting chicken MDA5 and IFN-β to knock-down the expression of MDA5 and IFN-β. Cells were then stimulated with poly(I:C) for 12 h at a concentration of 1 μg/ml, and the mRNA levels of *CMPK2*, *IFN-β*, and *Mx1* were measured *via* qRT-PCR. The protein of CMPK2 was detected *via* WB. The gray intensity for each band was measured, and the relative ratio of CMPK2 was determined from the equation (Sample^CMPK2^/Sample^actin^)/(Mock^CMPK2^/Mock^actin^) and marked at the bottom of each lane. Values represent the mean of the individual measurements in each sample ± SEM. ^*^p < 0.05, ^**^p < 0.01, ^***^p < 0.001, and ^****^p < 0.0001.

According to previous studies, the upregulation of *CMPK2* mRNA can be induced by poly(I:C), LPS, and IFN-β in human and fish cells (Collins et al., 2007; El-Diwany et al., 2018; Kim et al., 2021). In the present study, we found that the *CMPK2* mRNA levels increased after poly(I:C) stimulation as well as upon virus infection, which was consistent with the increase of IFN-β levels (Figures 4C,D,H). To determine whether chCMPK2 expression could be regulated in response to IFN-β or IL stimulation, the *CMPK2* mRNA levels in DF-1 cells were detected after stimulation with plasmids expressing chicken IFN-α, IFN-β, IFN-γ, IL-6, IL-8, and IL-1β. Compared to the results in mammals and fish, the *chCMPK2* mRNA level is a strong indicator of type I IFN and type III IFN levels, but shows a minimal response to IL stimulation (Figure 6A). To determine whether the expression of chCMPK2 was regulated by MDA5 and IFN-β, DF-1 cells were stimulated with specific siRNA targeting MDA5 or IFN-β for 24 h and then treated with poly(I:C). The effect of siRNAs was tested by qRT-PCR (Supplementary Figure S2). Both mRNA and protein-expression levels of chCMPK2 were markedly reduced after siRNA-mediated knockdown of MDA5 or IFN-β and poly(I:C) treatment (Figure 6B). All these data indicated that chicken CMPK2 was associated with MDA5 and IFN-β expression. Consistent with previous findings in human, these data confirmed that *chCMPK2* was an MDA5/IFN-β-inducible gene in chicken cells.

### Antiviral Effects of chCMPK2 on AIV and NDV

**Figure 7:**
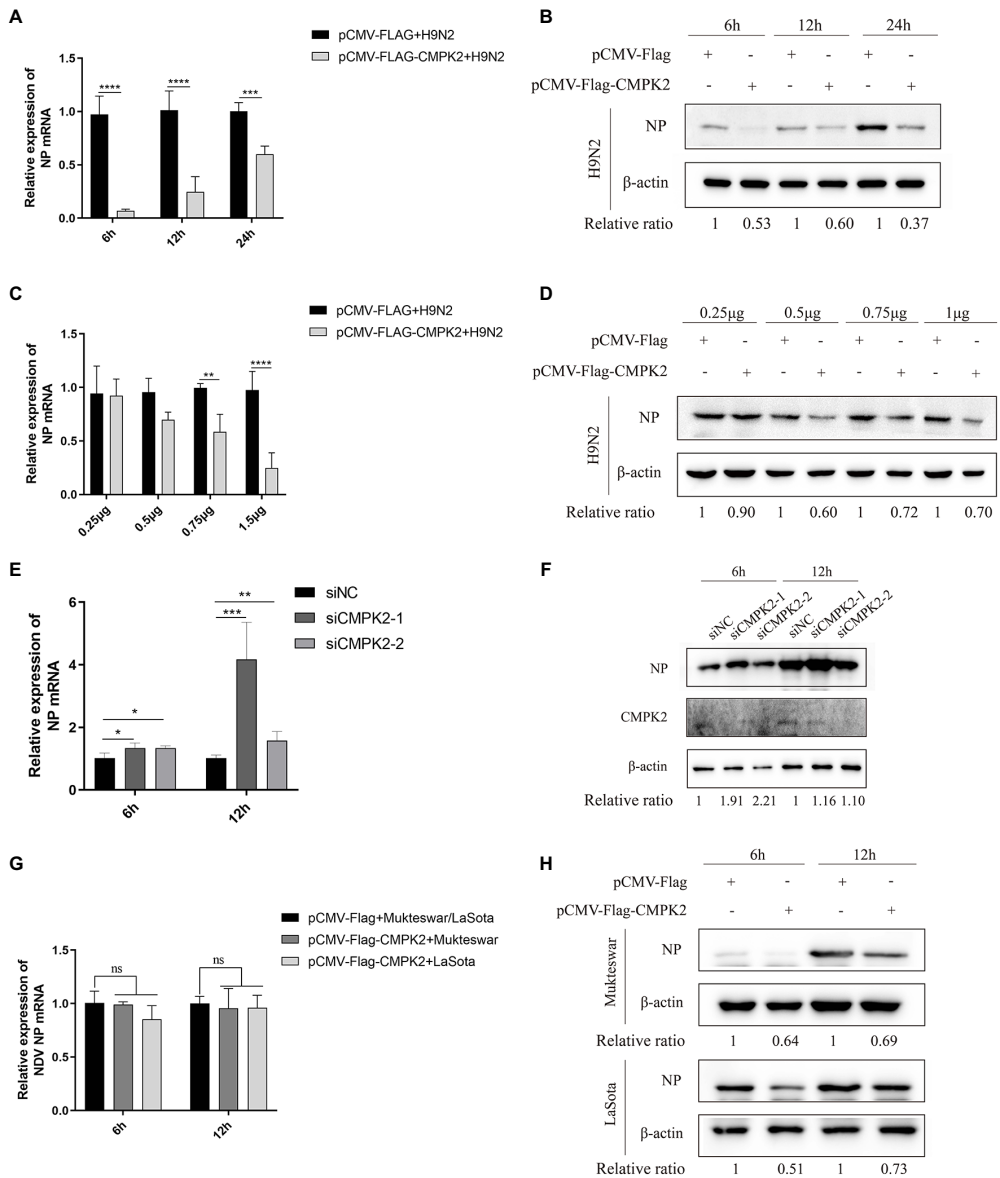
Antiviral effects of chCMPK2 on AIV and NDV. DF-1 cells were transfected with pCMV-Flag-CMPK2 or pCMV-Flag (control) for 24 h and then infected with AIV H9N2 at an MOI of 0.1 for 6, 12, and 24 h. The expression levels of H9N2 NP were detected by qRT-PCR **(A)** and WB **(B)** assays. DF-1 cells were transfected with pCMV-Flag-CMPK2 at different doses (0.25, 0.5, 0.75, and 1.5 μg) for 24 h and then infected with H9N2 at an MOI of 0.1 for 6 h. The expression levels of H9N2 NP were detected by qRT-PCR **(C)** and WB **(D)** assays. DF-1 cells transfected with CMPK2 siRNAs or siRNA-NC were infected with H9N2 at an MOI of 0.1. After 6 and 12 h, H9N2 viral NP levels in infected DF-1 cells were measured by qRT-PCR **(E)** and WB **(F)**. DF-1 cells were transfected with pCMV-Flag-CMPK2 or pCMV-Flag (control) for 24 h and then infected with NDV strains LaSota and Mukteswar at an MOI of 0.1 for 6 and 12 h. The expression levels of NDV NP were detected by qRT-PCR **(G)** and WB **(H)**. The gray intensity for each band was measured, and the relative ratio of AIV NP was determined from the equation (Sample^NP^/Sample^actin^)/(Mock^NP^/Mock^actin^) and marked at the bottom of each lane. Values represent the mean of the individual measurements in each sample ± SEM. ^*^p < 0.05, ^**^p < 0.01, ^***^p < 0.001, and ^****^p < 0.0001.

In SVCV-infected FHM cells, overexpression of CMPK2 has significant antiviral effects. The opposite effects were observed when CMPK2 was knocked-down (Liu et al., 2019). To further explore the role of *chCMPK2* in antiviral immune responses, pCMV-Flag-CMPK2 or pCMV-Flag (control) was transfected into DF-1 cells, and at 24 h post-transfection the cells were infected with AIV H9N2 at an MOI of 0.1. Then qRT-PCR and WB analysis were performed and the results showed that overexpression of CMPK2 significantly suppressed AIV H9N2 replication compared with the Flag control at both mRNA and protein levels at 6, 12, and 24 hpi (Figures 7A,B). Furthermore, it is noted that the antiviral effect of chCMPK2 appeared to be in a dose-dependent way (Figures 7C,D).

Further, *chCMPK2* gene was knocked down in DF-1 cells by transfection with CMPK2-specific siRNA, the knockdown efficiency of gene transcription was approximately 60%, and the expression level was approximately 70% lower (Supplementary Figure S2C). To obtain more functional evidence for chCMPK2 in antiviral responses, DF1 cells were treated with chCMPK2 siRNA followed by transfection with AIV H9N2. As shown in Figures 7E,F, viral NP protein increased in chCMPK2 knockdown cells compared to control cells in 6 and 12 hpi, and which were consistent with the results of qRT-PCR. Together, these results demonstrated that chCMPK2 was required for host defense against AIV H9N2 infection *in vitro*.

In order to determine the antiviral effect of chCMPK2 on NDV, DF-1 cells were infected with LaSota and Mukteswar at an MOI of 0.1 after transfection with chCMPK2 for 24 h. There was no significant change in the mRNA levels of NDV NP (Figures 7G,H) at 6 and 12 dpi. However, the viral NP protein levels of LaSota and Mukteswar were significantly reduced at both 6 and 12 dpi. These data indicated that chCMPK2 may be also an antiviral molecule against NDV.

### The TMK Domain Plays an Important Role in the Antiviral Effect of chCMPK2

**Figure 8:**
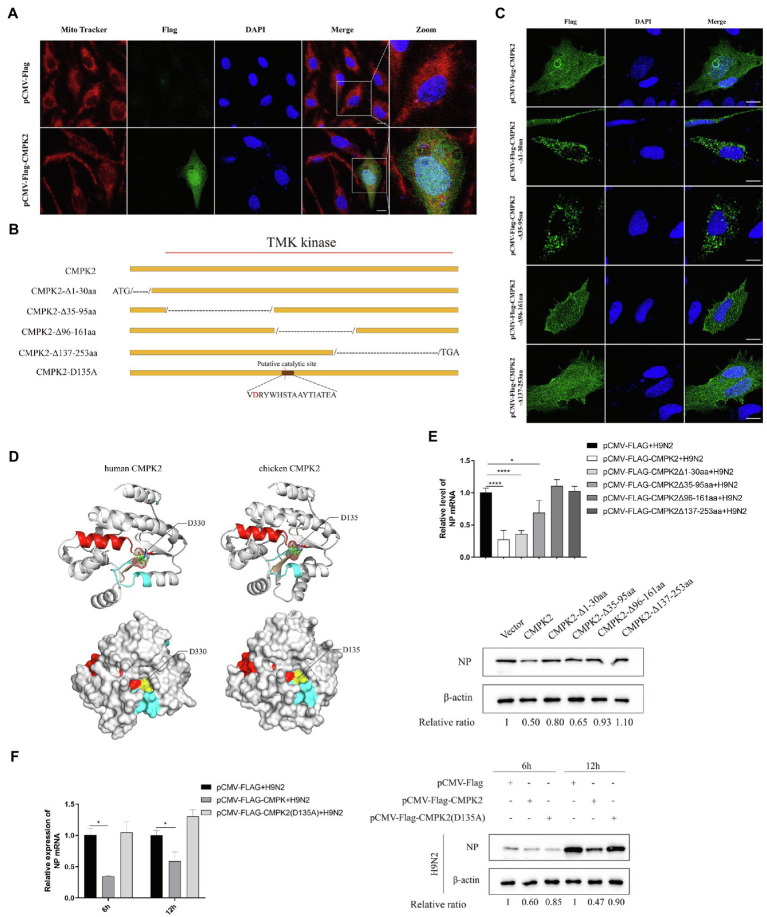
The role of the TMK domain in the antiviral effect of chCMPK2. **(A)** Subcellular localization of CMPK2 in DF-1 cells. DF-1 cells were transfected with pCMV-Flag control and pCMV-Flag-CMPK2. At 24 h post-transfection, mitochondria were stained with Mito Tracker Red for 30 min, cell nuclei were stained with DAPI, and samples were observed by confocal laser scanning microscopy. **(B)** The chCMPK2 was truncated into four parts (CMPK2-Δ1-30aa: amino acids 1–30 were truncated; CMPK2-Δ35-95aa: amino acids 35–95 were truncated; CMPK2-Δ96-161aa: amino acids 96–161 were truncated; CMPK2-Δ137-253aa: amino acids 137–253 were truncated) and the mutant plasmid chCMPK2(D135A) was designed to investigate the key fragments in antiviral function. **(C)** DF-1 cells were transfected with CMPK2-Δ1-30aa, CMPK2-Δ35-95aa, CMPK2-Δ96-161aa, CMPK2-Δ137-253aa, and pCMV-Flag (control) for 24 h, and then cells were stained with DAPI and samples were observed under by confocal laser scanning microscopy. **(D)** The human CMPK2 protein structure was obtained from the AlphaFold Protein Structure Database. The chicken CMPK2 protein structure was predicted by using huCMPK2 as a template. The P-loop, the putative catalytic site, and the lid motif are shown in yellow, red, and blue, respectively. D330 of huCMPK2 and D135 of chCMPK2 are labeled in the structures. **(E)** DF-1 cells were transfected with four truncated plasmids, pCMV-Flag-CMPK2 (positive control), and pCMV-Flag (negative control) for 24 h, and then cells were infected with H9N2 at an MOI of 0.1. At 6 hpi, the cells were collected to detect the expression of NP by qRT-PCR (up) and WB (down) assays. The gray intensity for each band was measured, and the relative ratio of AIV NP was determined from the equation (Sample^NP^/Sample^actin^)/(Mock^NP^/Mock^actin^) and marked at the bottom of each lane. **(F)** DF-1 cells were transfected with pCMV-Flag-CMPK2, pCMV-Flag, and CMPK2(D135A) and then infected with H9N2 at an MOI of 0.1. The expression of NP was measured by qRT-PCR (up) and WB (down). Values represent the mean of the individual measurements in each sample ± SEM. ^*^p < 0.05 and ^****^p < 0.0001.

To determine the subcellular localization of CMPK2, pCMV-Flag-CMPK2 was transfected into DF-1 cells. The high expression of CMPK2 in transfected DF-1 cells was verified by WB (Supplementary Figure S1D), and the fluorescence was observed under a confocal microscope. As shown in Figure 8A, the green fluorescence of the CMPK2 fusion protein was distributed in the cytoplasm and nucleus of DF-1 cells. In previous reports, human CMPK2 and fish CMPK2 was localized in the mitochondria of HeLa cells and FHM cells (Xu et al., 2008; Liu et al., 2019). In the present study, mitochondria were stained by Mito Tracker, and chicken CMPK2 was partially localized in the mitochondria of DF-1 cells.

To determine the key domain of chCMPK2 for its antiviral effect against AIV H9N2, full-length chCMPK2 was truncated in four ways (Figure 8B). DF-1 cells were transfected with four truncated chCMPK2 mutant plasmids to identify the antiviral effects and the changes in subcellular localization were observed. As shown in Figure 8C, there were no influences on the subcellular localization of chCMPK2 after the C-terminal 159 amino acids were truncated (CMPK2-Δ96-161aa and CMPK2-Δ137-253aa), while no nuclear localization was observed when the N-terminal region was truncated (CMPK2-Δ1-30aa and CMPK2-Δ35-95aa). It is inferred that the nuclear localization signal sequence was located in the first 95 amino acids on the N-terminus of chCMPK2. However, the nuclear localization of chCMPK2 was not related to its antiviral effect, because CMPK2-Δ1-30aa and CMPK2-Δ35-95aa showed similar antiviral activity against AIV with that of full-length chCMPK2. As shown in Figure 8E, the antiviral effects of CMPK2-Δ96-161aa and CMPK2-Δ137-253aa were significantly inhibited, suggesting the fragments determining the antiviral function of chCMPK2 were located at the C-terminus of chCMPK2, which consisted of an evolutionarily conserved TMK domain.

To identify the key amino acid for the function of TMK domain, the 3D models of the chCMPK2 and huCMPK2 TMK domains were constructed using homology modeling on the Swiss-model website. The positions of the three catalytic regions (P-loop, catalytic site, and lid domain) are consistent with those of humans (Figure 8D). The alignments revealed that the conserved putative catalytic site motif of chCMPK2 contained the highly conserved Asp135, Arg136, Trp138, His139, Ser140, Thr141, and Ala142 residues (Figure 2B). Previous studies of mammalian CMPK2 indicated that the highly conserved aspartate (D) residue in its catalytic pocket, which is located in the putative catalytic site, could determine the catalytic activity of huCMPK2 (Zhong et al., 2018). The catalytic activity and the activation of NLRP3, which was induced by LPS-stimulated mtDNA replication, could be inhibited by replacing the highly conserved aspartate (D) residue with alanine (A) (Zhong et al., 2018). According to the amino acid sequence alignment results above (Figure 2B; Table 3), chCMPK2 shows the same highly conserved aspartate residue at the 135th site, and chCMPK2 3D models show that chCMPK2 D135 residues are located in a pocket which coincides with the position of the huCMPK2 D330 residue (Figure 8D). To further investigate the role of the Asp135 site in the antiviral function of chCMPK2, a mutant plasmid named CMPK2 (D135A), in which Asp135 was replaced with alanine (A), was generated and transfected into DF-1 cells. The mutant showed significantly weaker effects on AIV H9N2 replication than wild-type chCMPK2 in mRNA and protein expression levels (Figure 8F). Taken together, our results indicated that the antiviral function of chCMPK2 was dependent on the catalytic activity of the TMK domain.

## Discussion

In early host antiviral responses, IFN plays a critical role *via* triggering signaling cascades to induce multiple ISGs, such as murine myxovirus resistance 1 (Mx1), IFITM proteins, tripartite motif (TRIM) family proteins, and viperin. ISGs could intervene with the virus life cycle at different stages to affect viral replication. As an ISG, *CMPK2* was co-transcribed with viperin in response to IFN stimulation after virus stimulation (Kambara et al., 2014; Gizzi et al., 2018). Furthermore, CMPK2 could increase the local concentration of cytidine triphosphate (CTP) for viperin to use as substrate to enhance the production of 3′-deoxy-3′,4′-didehydro-CTP (ddhCTP), which is necessary for the antiviral function of viperin during viral infection and inhibited SARS-CoV-2 polymerase activity in Huh7-hACE2 cells (Minton, 2018; Rivera-Serrano et al., 2020; Wood et al., 2021). However, the expression of chicken CMPK2 as well as its role in immunity is still unclear. In the present study, chicken CMPK2 was cloned and characterized, and its potential role upon AIV H9N2 infection has been assessed. We aligned the chicken CMPK2 amino acid sequence with CMPK2 sequences from other species, and the results showed high amino acid homology between birds and reptiles. The chicken *CMPK2* gene was located in chromosome 3 of the chicken genome, adjacent to but in the opposite direction of viperin, suggesting that they can be co-transcribed in response to IFN stimulation after virus infection, similar to human *CMPK2*. TMK belongs to the NMP kinase superfamily, and is widely present in bacteria, viruses, and mammals. Previous studies demonstrated that the highly conserved P-loop motif in TMK could bind to and position the α- and β-phosphoryl groups of ATP, which act as phosphoryl donors (Ostermann et al., 2000; Lavie and Konrad, 2004). Any mutations in the P-loop motif may abolish the activity of the TMK in yeast, human, or *E. coli* (Lavie et al., 1997; Brundiers et al., 1999). The second critical domain, catalytic site motif, plays an important role in phosphorylation. Asp96 in human TMK is necessary for the binding to and positioning of the magnesium ion complexed to ATP, and Arg97 can promote the interaction between the γ-phosphate of ATP and the phosphate group of TMP (Xu et al., 2008). The third domain Lid-region is a flexible stretch which involved in the process of stabilizing binding to ATP (Lavie and Konrad, 2004). To investigate the conservation of the TMK domain, the sequences of the TMK domain were aligned. We found that CMPK2 is highly conserved from fish to humans, the TMK domain of chCMPK2 is 70.74% identical in amino acid sequence to that of *H. sapiens*, and three catalytic regions (P-loop, catalytic site, and lid domain) were almost identical. However, the N-terminal amino acid sequence varied greatly between species, which may indicate that the function of the N-terminus was not conserved.

In this study, we found that infection with several viruses or treatment with poly(I:C) could substantially stimulate the expression of chCMPK2 at the mRNA and protein levels in different chicken cells (DF-1 cells, HD11 cells, and CEFs), which suggested that chCMPK2 played an important role in the innate immune response. Interestingly, *chCMPK2* mRNA expression increased before 12 hpi and decreased at 18 and 24 hpi upon AIV H9N2 infection. This finding was consistent with the *CMPK2* mRNA levels observed in THP-1, TZM-bl, and MT4 cells upon stimulation with IFN, though it differed from those in SVCV-infected FHM cells (El-Diwany et al., 2018; Liu et al., 2019). The reason may be that the host immune response is often different among cell types or viruses (Kang et al., 2020).

In the present study, MDA5 and IFN-β were found to be involved in the production of chCMPK2 upon virus infection. Our results were consistent with preliminary hypothesis on duck that CMPK2 may be induced through the IFNα/β signaling pathway (Xiang et al., 2020). MDA5 and RIG-I, as members of the evolutionarily conserved RIG-like helicase family, play critical roles in the host antiviral defense against pathogens. Chicken has high susceptibility to AIV due to the lack of RIG-I (Barber et al., 2010). Chicken MDA5 (chMDA5) acts as another viral RNA recognition receptor, which is involved in the type I IFN responses induced by NDV, AIV, or poly(I:C; Cheng et al., 2015b; Yu et al., 2020). After invasion by foreign microorganisms, chMDA5 recognizes the double-stranded RNA in the cytoplasm together with the mitochondrial antiviral signaling (MAVS) and activates a cascade with other adaptor molecules such as IKKε and TANK binding kinase 1 (TBK1; Jia et al., 2021). Then the signal activates IRF3 by phosphorylation, followed by IFN synthesis (Fitzgerald et al., 2003; Ojha et al., 2021). Upon the synthesis and secretion of IFN, which binds to cell surface receptors, the signal is transmitted through the membrane and into the cell, and subsequently ISGs are stimulated, inhibiting viral replication through the JAK/STAT signaling pathway (Schneider et al., 2014). Chicken MDA5 can partially rescue the compromised viral RNA recognition in the absence of RIG-I and preserves antiviral competence (Xu et al., 2019; Krchlikova et al., 2021). In the present study, we found that IFN-β levels showed a similar pattern as chCMPK2 at 6, 12, 18, and 24 h after infection with AIV H9N2 and NDV. Though CMPK2 has been identified as an ISG in human, little is known about its regulatory pathway. A recent study revealed that CMPK2 expression is dependent on LPS/poly(I:C)-mediated IRF3 type I IFN signaling by inhibiting the IFN-α receptor (IFNAR) in THP-1 cells and bone marrow-derived macrophages (BMDMs) derived from IFNAR knockout and IRF3 knockout mice (Kim et al., 2021). According to our results, IFNs are strong inducers of chCMPK2 expression, while IL had little influence on it. chCMPK2 expression was significantly inhibited when MDA5 or IFN-β was defective in DF-1 cells. Taken together, our results suggested that chCMPK2 was regulated by the MDA5/IFN-β signaling pathway.

In the current report, we first demonstrated that chCMPK2 inhibited the propagation of AIV H9N2 in a dose-dependent way, and chCMPK2 may also be an antiviral molecule against NDV. CMPK2 has been reported to be able to inhibit viral and bacterial infection, including SVCV, Dengue virus (DENV), Duck Tembusu virus (DTMUV), HIV-1 and *Aeromonas hydrophila* CCL1 (El-Diwany et al., 2018; Liu et al., 2019; Xiang et al., 2020; Feng et al., 2021; Lai et al., 2021). Our analysis indicated that overexpression of chCMPK2 in DF-1 cells significantly inhibited H9N2 NP expression at the mRNA and protein levels, and knockdown of chCMPK2 had the opposite effects, which suggested that chCMPK2 was an important antiviral factor against AIV. Unexpectedly, NDV LaSota and Mukteswar NP proteins can be strongly suppressed after chCMPK2 overexpression, whereas there was no statistical difference in NP mRNA level. We speculate that chCMPK2 has an inhibitory effect on the translation process rather than the transcription process of NDV. Therefore, further experimental evidence is required to resolve the anti-NDV mechanism of chCMPK2.

Different subcellular localizations may yield different functions. As previously reported, CMPK2 localization showed differences between species and cell types. CMPK2 localized in the mitochondria of HeLa cells, whereas it was distributed in the cytoplasm and partially in the mitochondria of THP-1 cells (Kim et al., 2021). In FHM cells, CMPK2 is localized in the cytoplasm and displays colocalization with mitochondria (Chen et al., 2008; Xu et al., 2008). Interestingly, in DF-1 cells, CMPK2 was not only located in mitochondria but also in nuclei. Xu et al. (2008) proposed that the mitochondrial targeting signal was located in the first 22 amino acids. We truncated the first 30 amino acids, resulting in the loss of nuclear localization, indicating that the nuclear transport signal of chCMPK2 was indeed present at the N-terminus. We suspected that the large differences in N-terminal amino acid sequence between species led to the differences in subcellular localization. The antiviral activity of viperin was found to be dependent on the C-terminal region of the protein, while it was not associated with the co-localization with lipid droplets (Helbig et al., 2013). In the present study, truncation in the N-terminal region did not affect the anti-AIV H9N2 function of chCMPK2, as determined by measuring the replication of AIV H9N2, which suggested that the N-terminus may not be the most important domain, that the antiviral activity of chCMPK2 resided in its C-terminal region, and that the antiviral activity of chCMPK2 did not rely on its nuclear localization.

In the present study, the TMK domain and the D135 key catalytic site were proven to be linked to the antiviral effect of chCMPK2. The TMK domain, which is located in the C-terminus of CMPK2, is known as a nucleoside monophosphate kinase that catalyzes the formation of dTDP by transferring phosphoryl from ATP to deoxythymidylic monophosphate (dTMP) in a Mg^2+^-dependent manner using ATP as the phosphoryl donor (Li de la Sierra et al., 2001; Aufderklamm et al., 2012; Doharey et al., 2014). In addition to its vital role in supplying precursors for DNA synthesis, the catalytic activity of TMK has an important role in the activation of NLRP3, which is activated by LPS-induced mtDNA synthesis in mouse BMDMs. In other studies of the antiviral effects of ISGs, the enzymatic activity of viperin was directly linked to its antiviral mechanism due to the production of ddhCTP, representing the only activity of viperin in human cells (Rivera-Serrano et al., 2020). The enzymatic activity of chCMPK2 still is connected with its antiviral effect. A mutation in the key catalytic site of the TMK domain prompted us to further investigate its function (Zhong et al., 2018). In the present study, mutation of the Asp135 residue caused chCMPK2 to lose its antiviral function during AIV H9N2 infection. Thus, the antiviral activity of chCMPK2 depended on the activity of its C-terminus.

According to previous researches, CMPK2 plays an important role in the production of dTTP and keeping low intra-cellular dUTP/dTTP ratios, which could subsequently prevent dUTP insertion into viral genome and weaken the dUTP caused impairment to virus replication (Daikoku et al., 1991; Voronin et al., 2014; Wang et al., 2020). Furthermore, pyrimidine analogs, deoxycytidine analogs and deoxyuridine analogs, including ddC, BVDU, were widely used in antiviral strategies due to their triphosphate form could compete with its natural counterpart for incorporation into the DNA or RNA (Derissen and Beijnen, 2020). The replication of viruses could be inhibited by those pyrimidine analogs, which could be produced by CMPK2 from their monophosphate forms *via* phosphorylation reaction. Considering the role of CMPK2 in the activation of pyrimidine analogs from their monophosphate forms, we speculated that the antiviral effect of CMPK2 may be related to the pyrimidine analogs (Xu et al., 2008). However, the underlying mechanism of antiviral effects remains to be investigated.

In sum, chicken CMPK2 plays an important role in host immune responses against AIV H9N2 and NDV infection, and a key site on the TMK catalytic domain, Asp135, was identified as critical for the antiviral activities of chCMPK2. Detailed chCMPK2 bioactivity in immunity must be characterized in the future.

## Data Availability Statement

The original contributions presented in the study are included in the article/**Supplementary Material**, and further inquiries can be directed to the corresponding authors.

## Author Contributions

XL and XQ designed the experiments, analyzed the data, and wrote the manuscript. XL and YF performed the experiments. XQ, CD, CX, WL, LT, CS, TR, and YL gave suggestions during the experiments. XL and XQ revised the manuscript. All authors read and approved the final manuscript.

## Funding

This work was supported by the Natural Science Foundation of Shanghai (grant nos. 21ZR1476800 and 20ZR1469400) and the Foundation of Key Laboratory of Veterinary Biotechnology (grant no. shklab202001).

## Conflict of Interest

The authors declare that the research was conducted in the absence of any commercial or financial relationships that could be construed as a potential conflict of interest.

The reviewer XW declared a shared affiliation with the authors XL, YF, WL, LT, YS, CS, YL, CD, and XQ at the time of the review.

## Publisher’s Note

All claims expressed in this article are solely those of the authors and do not necessarily represent those of their affiliated organizations, or those of the publisher, the editors and the reviewers. Any product that may be evaluated in this article, or claim that may be made by its manufacturer, is not guaranteed or endorsed by the publisher.

## Footnotes

^1^ https://www.ebi.ac.uk/Tools/msa/clustalo/

^2^ https://www.alphafold.ebi.ac.uk/

^3^ http://swissmodel.expasy.org/

^4^ http://jvenn.toulouse.inra.fr/app/example.html

^5^ https://fiji.sc/

## Supplementary Material

SUPPLEMENTARY TABLE S1 Primers used for cloning.

SUPPLEMENTARY TABLE S2 Primers used for qRT-PCR.

SUPPLEMENTARY TABLE S3 The siRNA used in this study.

SUPPLEMENTARY FIGURE S1 Construction of pCMV-Flag-CMPK2 and validation of the specificity and activity of the polyclonal murine anti-CMPK2 antibody.

SUPPLEMENTARY FIGURE S2 Efficiency of siRNA on the expression of MDA5, IFN-β, and CMPK2 in DF-1 cells.
