# Supplementary material for: A Role for the Chicken Interferon-Stimulated Gene CMPK2 in the Host Response Against Virus Infection
Source: Front Microbiol. 2022 May 11;13:874331. doi: 10.3389/fmicb.2022.874331 (PMC9132166; doi:10.3389/fmicb.2022.874331)
Supplement: SUPPLEMENTARY TABLE S1 — Primers used for cloning. [file Table_1.docx]

**Supplementary Table 1 | Primers used for PCR cloning.**

| **Primer name** | **Sequence of Oligonucleotide (5´–3´)** |
| --- | --- |
| chCMPK2 F | CGGAATTCATGCTGCCAGGCTGCACCTCG |
| chCMPK2 R | CGGATCCCAAGGCACAGTGTTTTTTAATTAGTTGCAGCA |
| chCMPK2Δ1-30aa F | CGGAATTCATGTGCGCGGCGCGGAT |
| chCMPK2Δ1-30aa R | CGCGGATCCCAAGGCACAGTGTTTTTTAAT |
| chCMPK2Δ35-95aa F | GCGGCGCGGTGGAGGACAATATTTGATGAAGAGCC |
| chCMPK2Δ35-95aa R | TCCTCCACCGCGCCGCGCACTCCCGCAG |
| chCMPK2Δ96-161aa F | TCAGCCAGGATGCCGTGTATAAGTGGCCTGAAG |
| chCMPK2Δ96-161aa R | CGGCATCCTGGCTGATGCAAGCTGGCG |
| chCMPK2Δ137-253aa F | CGGAATTCATGCTGCCAGGCTGCA |
| chCMPK2Δ137-253aa R | CGCGGATCCCCTGTCTACAATCACAGGTG |
| chCMPK2(D135A) F | CCTGTGATTGTAG*C*CAGGTATTGGCATAGCACAGCTGCT |
| chCMPK2(D135A) R | CAATACCTG*G*CTACAATCACAGGTGCCTGAGTGGAT |
| chIFN-α F | GGTACCATGGCTGTGCCTGCAAG |
| chIFN-α R | CTTAAGGATTCACGCGCACAACGGAC |
| chIFN-β F | GGATCCGCCACCATGACTGCAAACCATCAGT |
| chIFN-β R | CTTAAGAGTGACAACAACTCTGCAAACCTAC |
| chIFN-γ F | GGATCCGCCACCATGACTTGCCAGAC |
| chIFN-γ R | CTTAAGAATCGTTAACGTAGAGGAGACT |
| chIL-6 F | GGATCCGCCACCATGAACTTCACCGAG |
| chIL-6 R | CTTAAGAGTCCGTGACTTTGAGGACCA |
| chIL-8 F | GGATCCGCCACCATGAACGGCAAGC |
| chIL-8 R | CTTAAGAGTGTCACCACGTAGTCTTA |
| chIL-1β F | GGATCCGCCACCATGGCGTTCGTT |
| chIL-1β R | CTTAAGAGTCGCGGGTGAATCGAACAT |
