# Supplementary material for: A Role for the Chicken Interferon-Stimulated Gene CMPK2 in the Host Response Against Virus Infection
Source: Front Microbiol. 2022 May 11;13:874331. doi: 10.3389/fmicb.2022.874331 (PMC9132166; doi:10.3389/fmicb.2022.874331)
Supplement: SUPPLEMENTARY TABLE S2 — Primers used for qRT-PCR. [file Table_2.docx]

**Supplementary Table 1 | Primers used for PCR cloning.**

| **Primer name** | **Sequence of Oligonucleotide (5´–3´)** |
| --- | --- |
| chCMPK2 F | CGGAATTCATGCTGCCAGGCTGCACCTCG |
| chCMPK2 R | CGGATCCCAAGGCACAGTGTTTTTTAATTAGTTGCAGCA |
| chCMPK2Δ1-30aa F | CGGAATTCATGTGCGCGGCGCGGAT |
| chCMPK2Δ1-30aa R | CGCGGATCCCAAGGCACAGTGTTTTTTAAT |
| chCMPK2Δ35-95aa F | GCGGCGCGGTGGAGGACAATATTTGATGAAGAGCC |
| chCMPK2Δ35-95aa R | TCCTCCACCGCGCCGCGCACTCCCGCAG |
| chCMPK2Δ96-161aa F | TCAGCCAGGATGCCGTGTATAAGTGGCCTGAAG |
| chCMPK2Δ96-161aa R | CGGCATCCTGGCTGATGCAAGCTGGCG |
| chCMPK2Δ137-253aa F | CGGAATTCATGCTGCCAGGCTGCA |
| chCMPK2Δ137-253aa R | CGCGGATCCCCTGTCTACAATCACAGGTG |
| chCMPK2(D135A) F | CCTGTGATTGTAG*C*CAGGTATTGGCATAGCACAGCTGCT |
| chCMPK2(D135A) R | CAATACCTG*G*CTACAATCACAGGTGCCTGAGTGGAT |
| chIFN-α F | GGTACCATGGCTGTGCCTGCAAG |
| chIFN-α R | CTTAAGGATTCACGCGCACAACGGAC |
| chIFN-β F | GGATCCGCCACCATGACTGCAAACCATCAGT |
| chIFN-β R | CTTAAGAGTGACAACAACTCTGCAAACCTAC |
| chIFN-γ F | GGATCCGCCACCATGACTTGCCAGAC |
| chIFN-γ R | CTTAAGAATCGTTAACGTAGAGGAGACT |
| chIL-6 F | GGATCCGCCACCATGAACTTCACCGAG |
| chIL-6 R | CTTAAGAGTCCGTGACTTTGAGGACCA |
| chIL-8 F | GGATCCGCCACCATGAACGGCAAGC |
| chIL-8 R | CTTAAGAGTGTCACCACGTAGTCTTA |
| chIL-1β F | GGATCCGCCACCATGGCGTTCGTT |
| chIL-1β R | CTTAAGAGTCGCGGGTGAATCGAACAT |

**Supplementary Table 2 | Primers used for qPCR**

| **Primer name** | **Forward primers (5´–3´)** | **Reverse primers (5´–3´)** |
| --- | --- | --- |
| MDA5 | TGAAAGCCTTGCAGATGACTTA | GCTGTTTCAAATCCTCCGTTAC |
| MX1 | AAGCCTGAGCATGAGCAGAA | TCTCAGGCTGTCAACAAGATCAA |
| IFN-β | CCTCAACCAGATCCAGCATT | GGATGAGGCTGTGAGAGGAG |
| CMPK2 | GGTGCTGGACATCCTGGAGAAGT | GCTGGCGGAGACCTTAACAGAAC |
| H9N2-NP | CCTGCTTGTGTGTACGGACT | GGCTGTTTTGAAGCAGACGG |
| LaSota-NP | CAACAATAGGAGTGGAGTGTCTGA | CAGGGTATCGGTGATGTCTTCT |
| Mukteswar-NP | ATGGGTCACGGTAGCAAAGG | ATTGCGCTCCTACATACGGG |
| β-actin | TATTGCTGCGCTCGTTGTTGAC | GATACCTCTTTTGCTCTGGGCTTC |

**Supplementary Table 2 | Primers used for siRNA**

| **Target gene** | **Name** | **Sequence of Oligonucleotide (5´–3´)** |
| --- | --- | --- |
| MDA5 | siMDA5 | UGAAGAACCUAGAGGGAUU |
| IFN-β | siIFN-β 1 | GCAUCCUCCAACACCUCUU |
| IFN-β | siIFN-β 2 | GCAAUGCUUCGUAAACCAA |
| CMPK2 | siCMPK2-1 | GUGGAGGACAAUAUUUGAU |
| CMPK2 | siCMPK2-2 | GCAUAGCACAGCUGCUUAU |
| CMPK2 | siCMPK2-3 | CAGCUUGUUUCGACAAAGA |

**
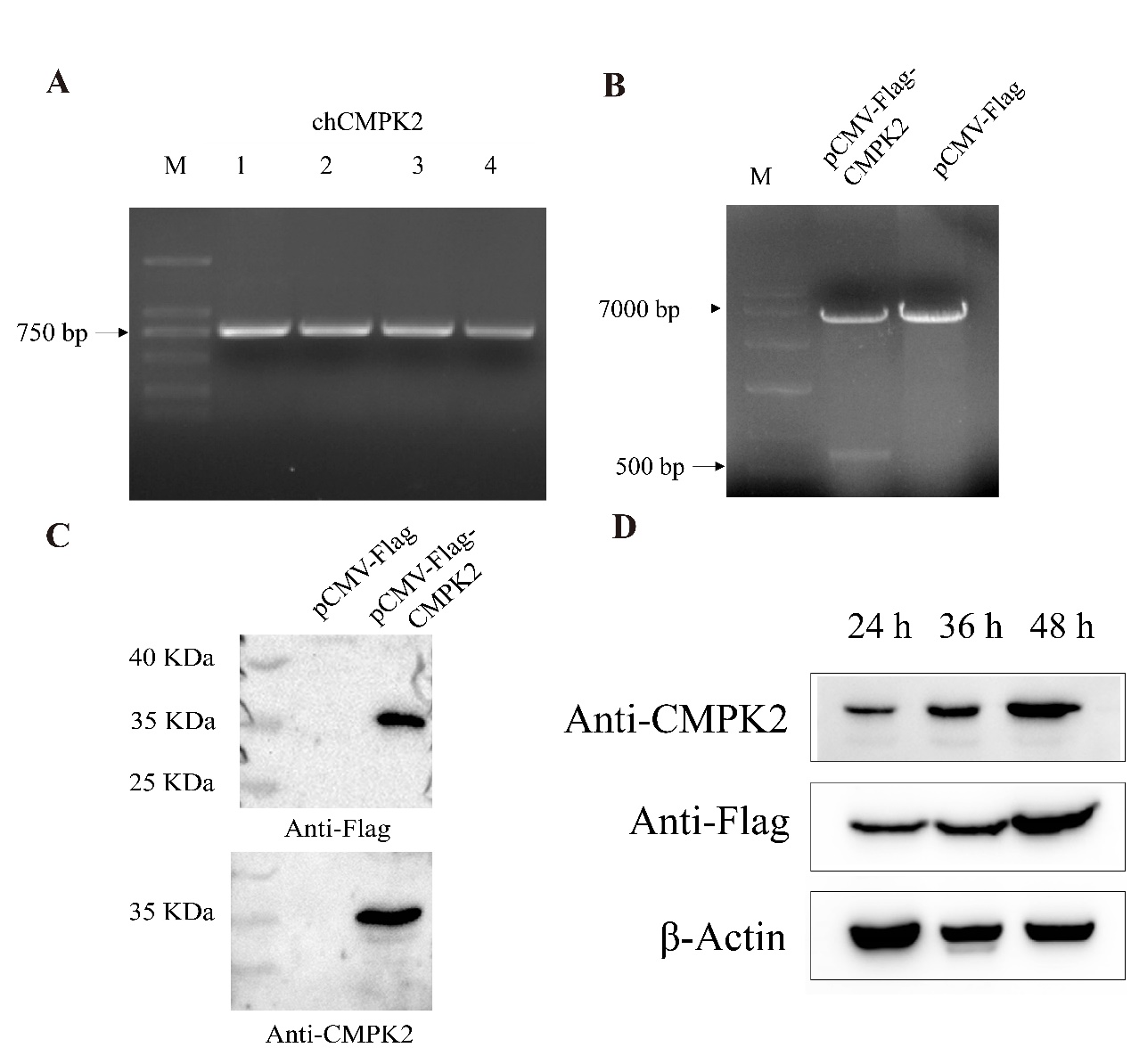
**

**FIGURE S1 |** Construction of pCMV-Flag-CMPK2 and validation of the specificity and activity of the murine polyclonal anti-CMPK2 antibody. (A) PCR amplification of the entire opening reading frame of the chicken *CMPK2* gene from DF-1 cells using the primer pairs chCMPK2_F and chCMPK2_R (Table S1). (B) PCR products were inserted into the pCMV-Flag vector through *EcoR*I and *BamH*I restriction enzyme sites. The ligation results were verified by double digestion with *EcoR*I and *BamH*I restriction sites. (C) HEK293T cells were transfected with pCMV-Flag-CMPK2 and pCMV-Flag (negative control) for 24 hours, and the cell lysates were used to detect the exogenous expression of chCMPK2 by Western blot analysis using anti-Flag and anti-CMPK2 to test the specificity of the polyclonal antibody. (D) HEK293T cells were transfected with pCMV-Flag-CMPK2 for 24, 36, and 48 hours to test the activity of the murine polyclonal anti-CMPK2 antibody.
