# Supplementary material for: A Role for the Chicken Interferon-Stimulated Gene CMPK2 in the Host Response Against Virus Infection
Source: Front Microbiol. 2022 May 11;13:874331. doi: 10.3389/fmicb.2022.874331 (PMC9132166; doi:10.3389/fmicb.2022.874331)
Supplement: SUPPLEMENTARY TABLE S3 — The siRNA used in this study. [file Table_3.docx]

**Supplementary Table 3 | Primers used for siRNA**

| **Target gene** | **Name** | **Sequence of Oligonucleotide (5´–3´)** |
| --- | --- | --- |
| MDA5 | siMDA5 | UGAAGAACCUAGAGGGAUU |
| IFN-β | siIFN-β 1 | GCAUCCUCCAACACCUCUU |
| IFN-β | siIFN-β 2 | GCAAUGCUUCGUAAACCAA |
| CMPK2 | siCMPK2-1 | GUGGAGGACAAUAUUUGAU |
| CMPK2 | siCMPK2-2 | GCAUAGCACAGCUGCUUAU |
| CMPK2 | siCMPK2-3 | CAGCUUGUUUCGACAAAGA |
