# Supplementary figures and images for: A Role for the Chicken Interferon-Stimulated Gene CMPK2 in the Host Response Against Virus Infection
Source: Front Microbiol. 2022 May 11;13:874331. doi: 10.3389/fmicb.2022.874331 (PMC9132166; doi:10.3389/fmicb.2022.874331)

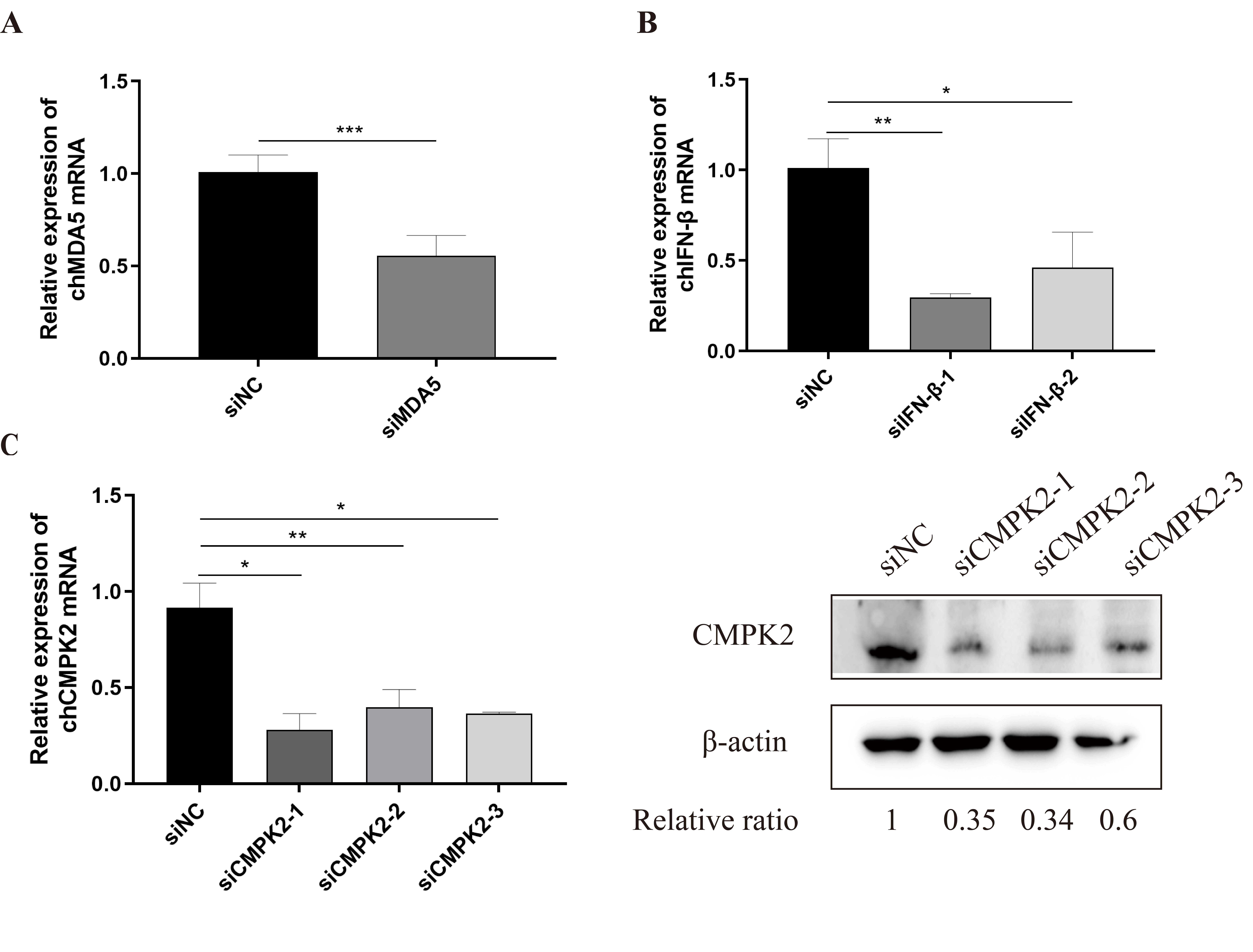

Supplement: SUPPLEMENTARY FIGURE S2 — Efficiency of siRNA on the expression of MDA5, IFN-β, and CMPK2 in DF-1 cells. [file Image_2.png]
